# Arthroscopic Technique for the Treatment of Localized Pigmented Villonodular Synovitis of the Knee

**DOI:** 10.7759/cureus.7832

**Published:** 2020-04-25

**Authors:** Yvonne-Mary Papamerkouriou, Markos I Posantzis, Dimitrios Kouremenos, Christos Manousakis, Spyridon I Plessas

**Affiliations:** 1 Orthopaedics, Panagiotis & Aglaia Kyriakou Children's Hospital, Athens, GRC; 2 Orthopaedics, General Hospital of Nikaia "Saint Panteleimon", Piraeus, GRC; 3 Orthopaedics, General Children's Hospital “Panagiotis & Aglaia Kyriakou”, Athens, GRC

**Keywords:** lpvns, arthroscopy, locking, knee

## Abstract

Pigmented villonodular synovitis (PVNS) is caused by a proliferation of the synovial membrane and can be a rare cause of pain and locking of the knee. In its localized form, it can be removed arthroscopically. We describe in detail a step-by-step arthroscopic technique applied to treat a 27-year-old patient who had been suffering from pain and episodes of locking for a year and whose left knee MRI revealed an intra-articular mass. The formation was completely enucleated arthroscopically and histological analyses confirmed the diagnosis of localized PVNS. There were no complications, and the patient was symptom-free at the six-month follow-up with no clinical or radiological evidence of recurrence.

## Introduction

Pigmented villonodular synovitis (PVNS) is a rare, benign condition, involving the proliferation of the synovial membrane, with an average annual incidence of 1.8 cases per 1 million population for intra-articular forms of the disease [1-3]. According to the current World Health Organization (WHO) classification, there are two main types of the disease: tenosynovial giant cell tumors, which are localized type (synonym: nodular tenosynovitis), and tenosynovial giant cell tumors, which are diffuse type (synonym: PVNS/tenosynovitis). The first type presents as a well-separated tumor surrounded by a fibrous capsule, whereas the second demonstrates infiltrative growth [2,4,5]. It is usually monoarticular, affecting adults in the third or fourth decade of life, with the knee being the most commonly involved anatomical location [6,7]. The lesion is composed of a mixture of mononuclear cells accompanied by multinucleated osteoclast-like giant cells and hemosiderin-laden macrophages [8]. Differential diagnosis includes synovial chondromatosis, rhabdomyosarcoma, fibroma of the tendon sheath, synovial sarcoma, amyloid arthropathy, hemophilic arthropathy, and lipoma arborescens. Clinical features of PVNS include pain, edema, joint dysfunction, and, rarely, a soft tissue mass [9]. Joint dysfunction may include knee locking, due to mechanical block, or pseudo-locking, due to a functional block. MRI is the modality of choice in PVNS diagnosing [4]. Arthroscopic excision is considered the gold standard in the treatment of localized PVNS (LPVNS) [10,11]. We describe the technique by which this is performed.

## Technical report

**Figure 1:**
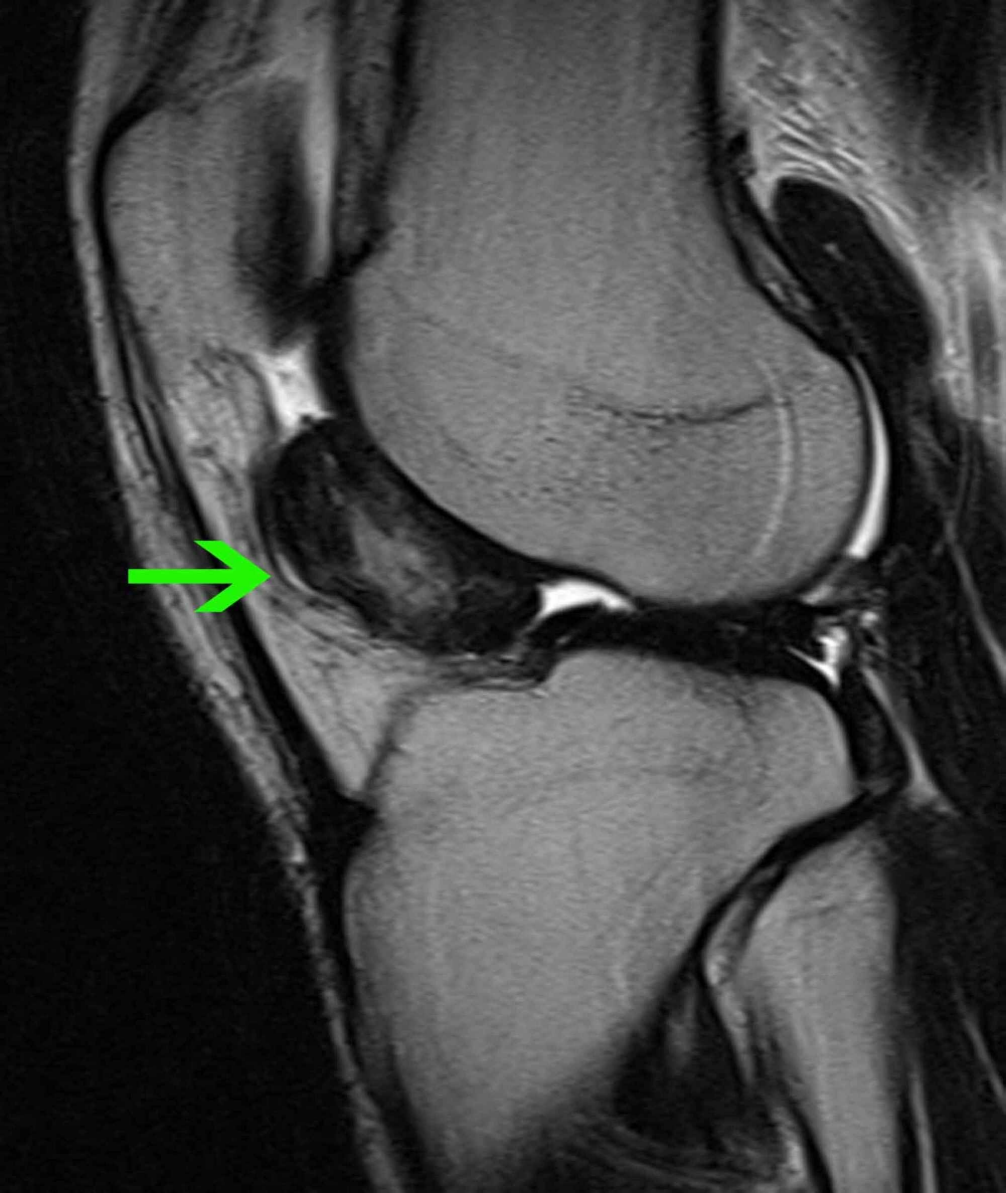
MRI of the left knee (sagittal plane) T2-weighted turbo spin-echo (TSE) sequence, sagittal plane, in which a heterogeneous signal mass can be seen posterior to the fat pad (green arrow).

**Figure 2:**
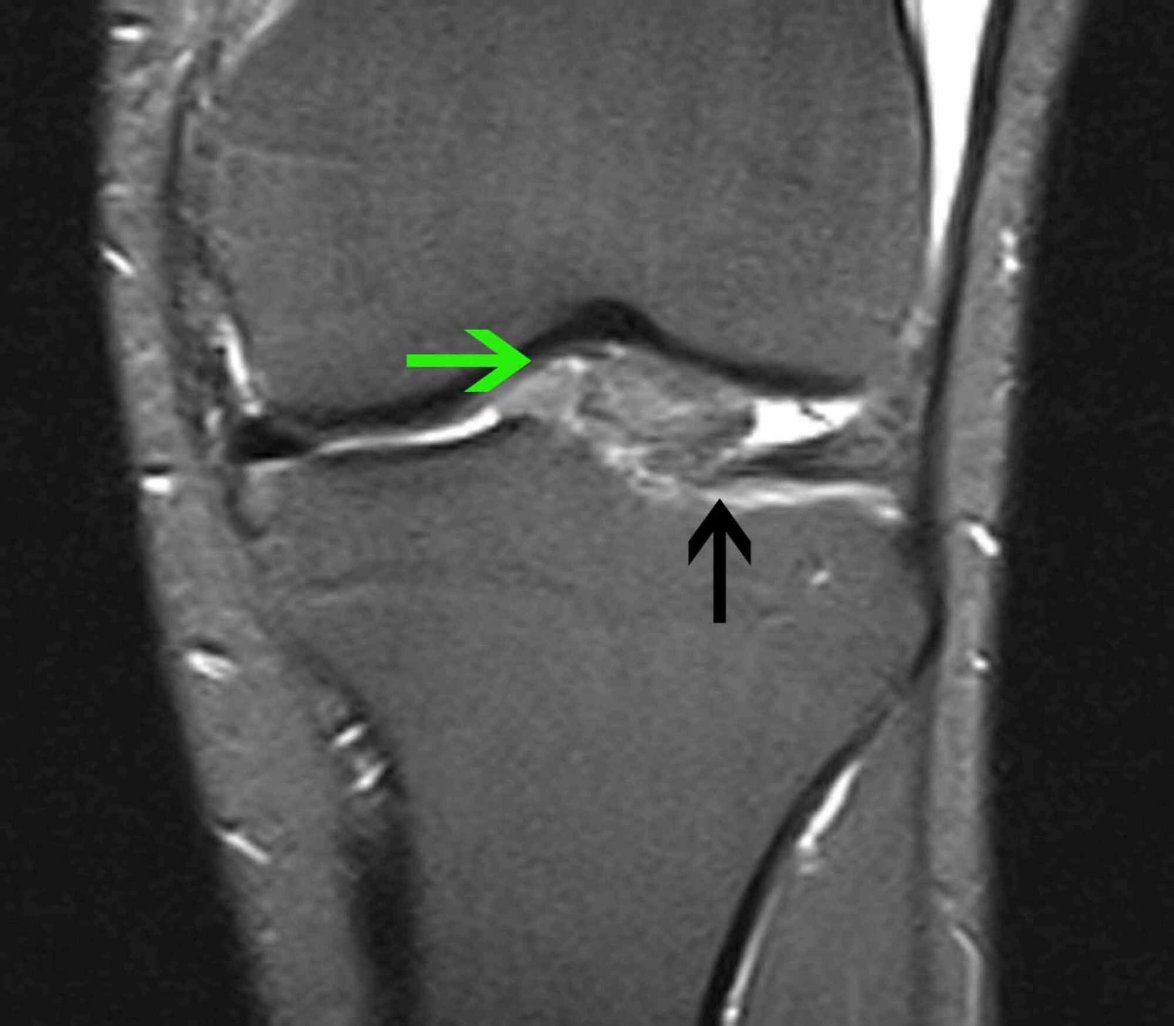
MRI of the left knee (coronal plane) T1-weighted turbo inversion recovery magnitude (TIRM) sequence, coronal plane, in which the mass can be seen between the lateral femoral condyle and the lateral tibial plateau (green arrow). It appears to be arising from the anterior attachment of the lateral meniscus (black arrow).

**Figure 3:**
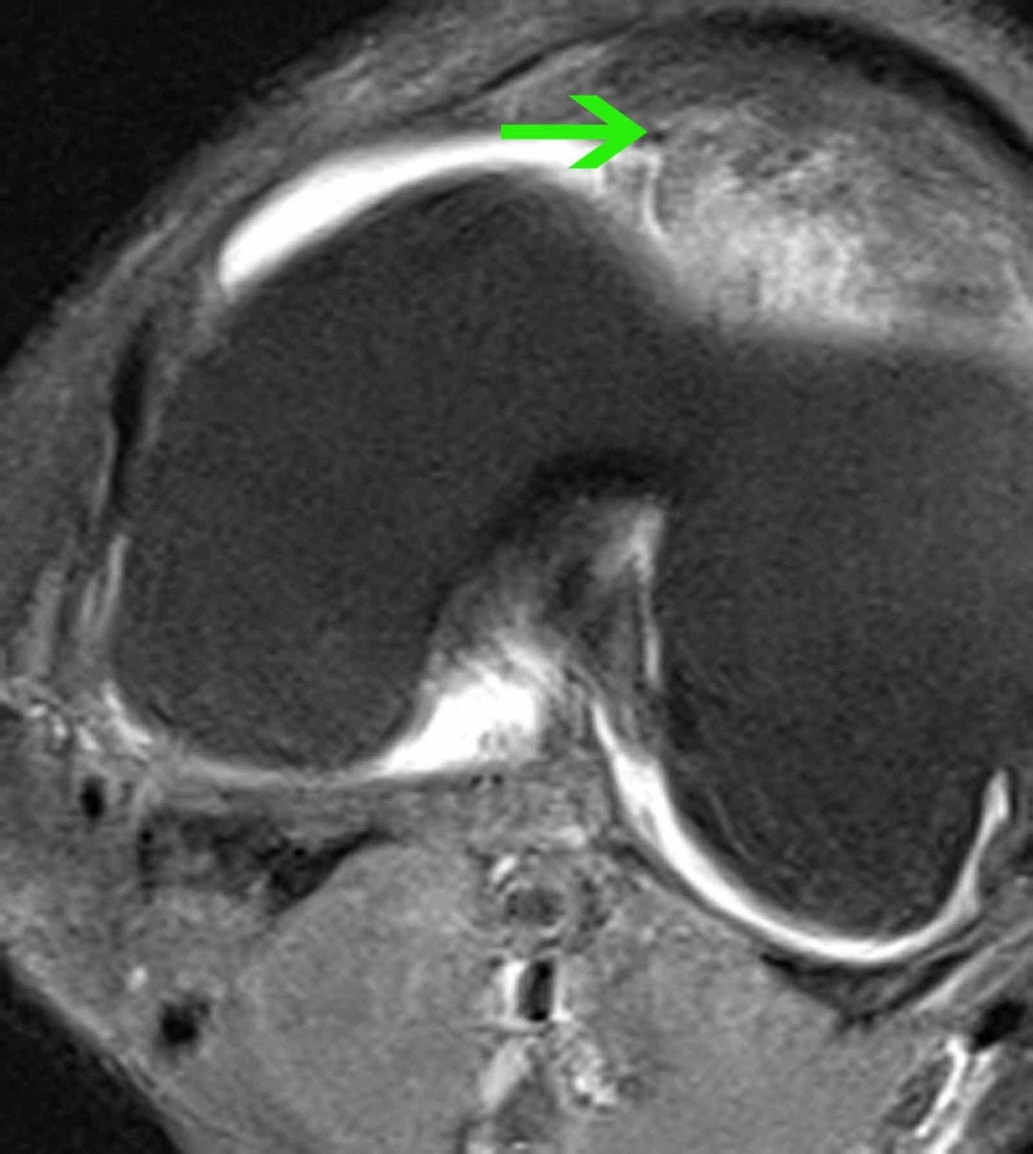
MRI of the left knee (transverse plane) Proton-density turbo spin-echo fat-suppressed (PD TSE FS) sequence, transverse plane, in which the mass is shown to be distal to the level of the patella behind the fat pad and anterior to the lateral femoral condyle (green arrow).

A 27-year-old male who had been suffering from ongoing pain in his left knee during the last year and had experienced at least two episodes of locking was referred to our orthopedic department. Initially, he had been treated conservatively with NSAIDs (nonsteroidal anti-inflammatory drugs) and physiotherapy. There was no specific history of injury. Clinical examination revealed a full range of motion (ROM), and there was no evidence of joint effusion. The patient had a mildly positive McMurray test on the medial joint line. In addition, there was tenderness of the patellofemoral joint. His X-rays appeared normal. MRI scan of the left knee without contrast revealed intact menisci, and there was no evidence of a loose body in the knee. However, there was a heterogeneous signal intensity mass posterior to the fat pad, seemingly arising from the anterior attachment of the lateral meniscus, including the transverse ligament (Figures 1-3).

**Figure 4:**
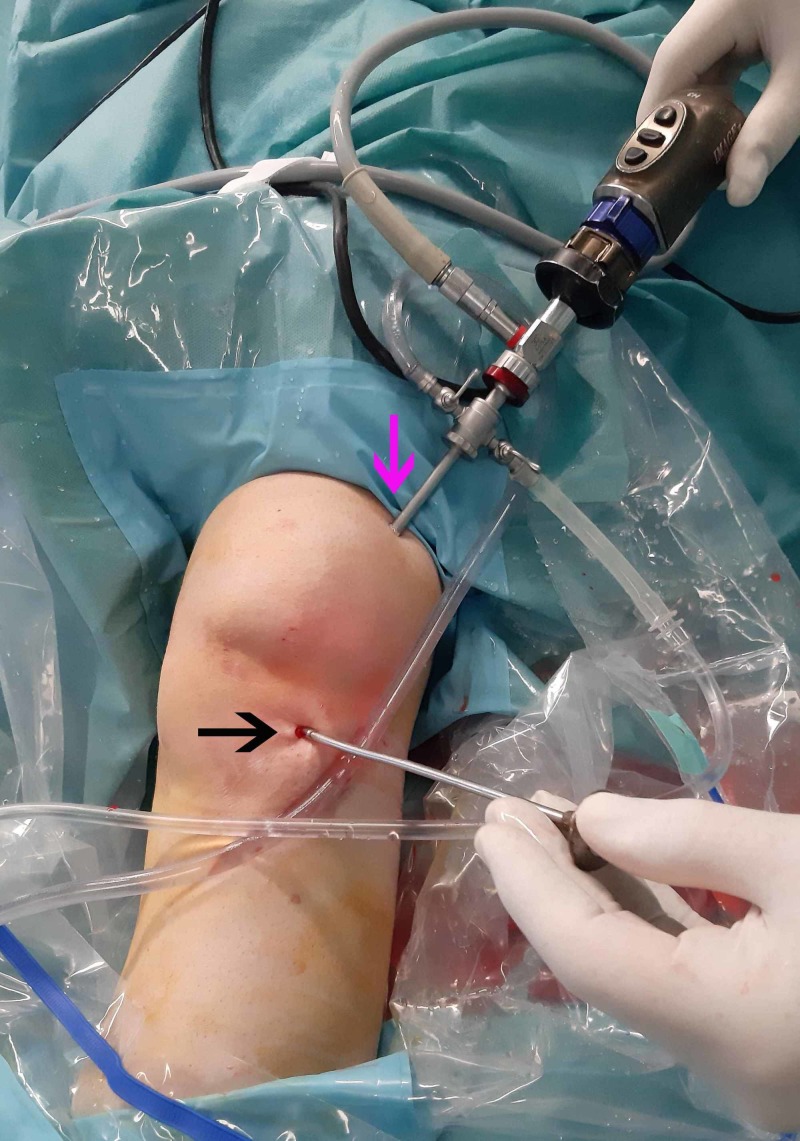
Superolateral and anterolateral arthroscopic portals Establishment of the initial superolateral portal (pink arrow) and through that creation of the anterolateral portal by direct vision. A blunt trocar was inserted through the anterolateral portal (black).

**Figure 5:**
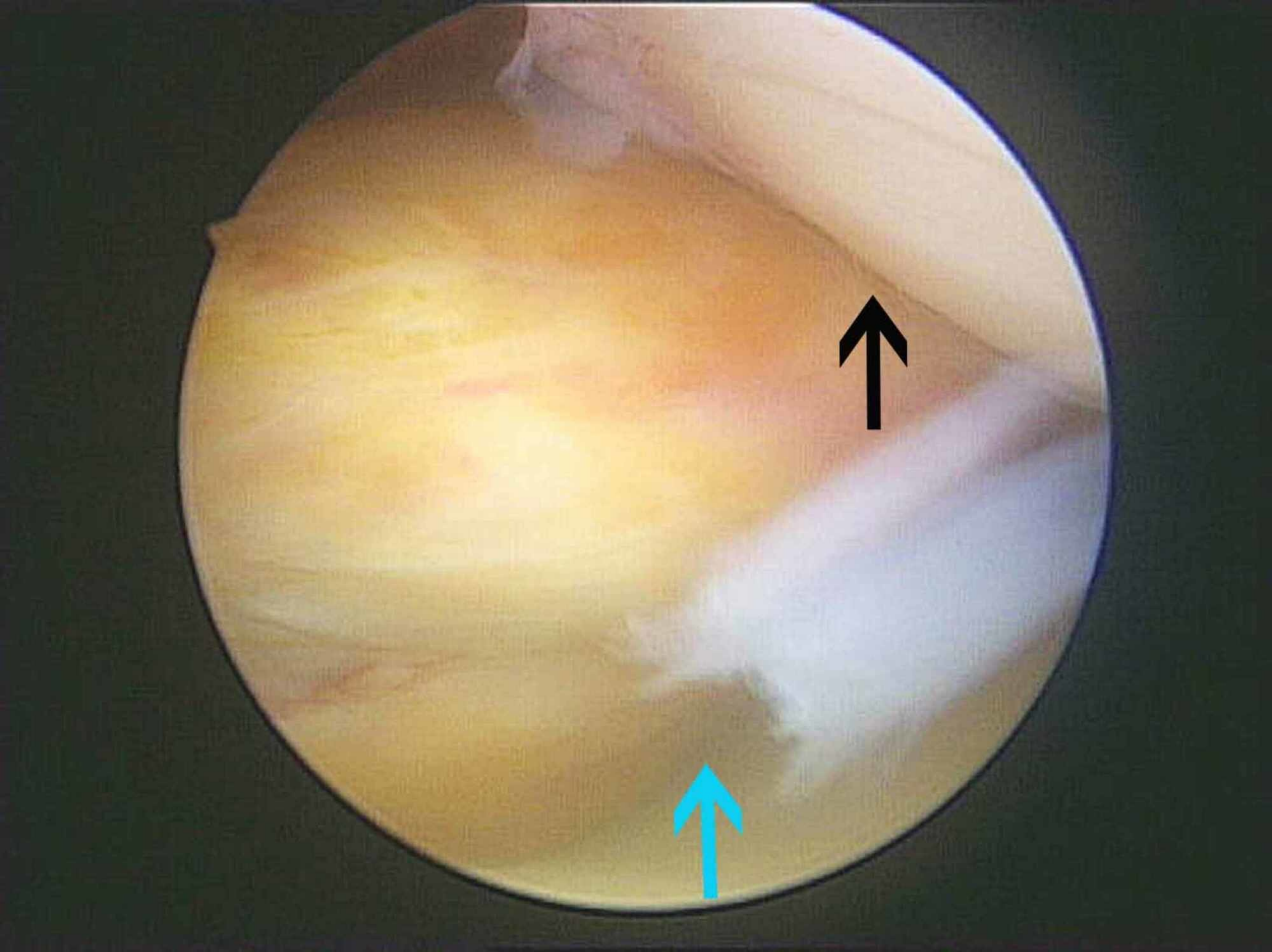
View from the superolateral portal View from the superolateral portal where the tumor appeared as a firm tan-yellow mass (blue arrow) and the patella was also apparent (black arrow).

The tumor did not have malignant features and, therefore, arthroscopic removal was decided upon. Firstly, the superolateral portal was established to view the tumor and estimate its nature. Under direct vision through the superolateral portal, the anterolateral portal was created. Using a blunt trocar, the location was assessed, as well as the origin of the tumor, specifically the anterior attachment of the lateral meniscus including the transverse ligament (Figure 4). Viewed from the superolateral portal, the tumor appeared as a firm tan-yellow mass (Figure 5).

**Figure 6:**
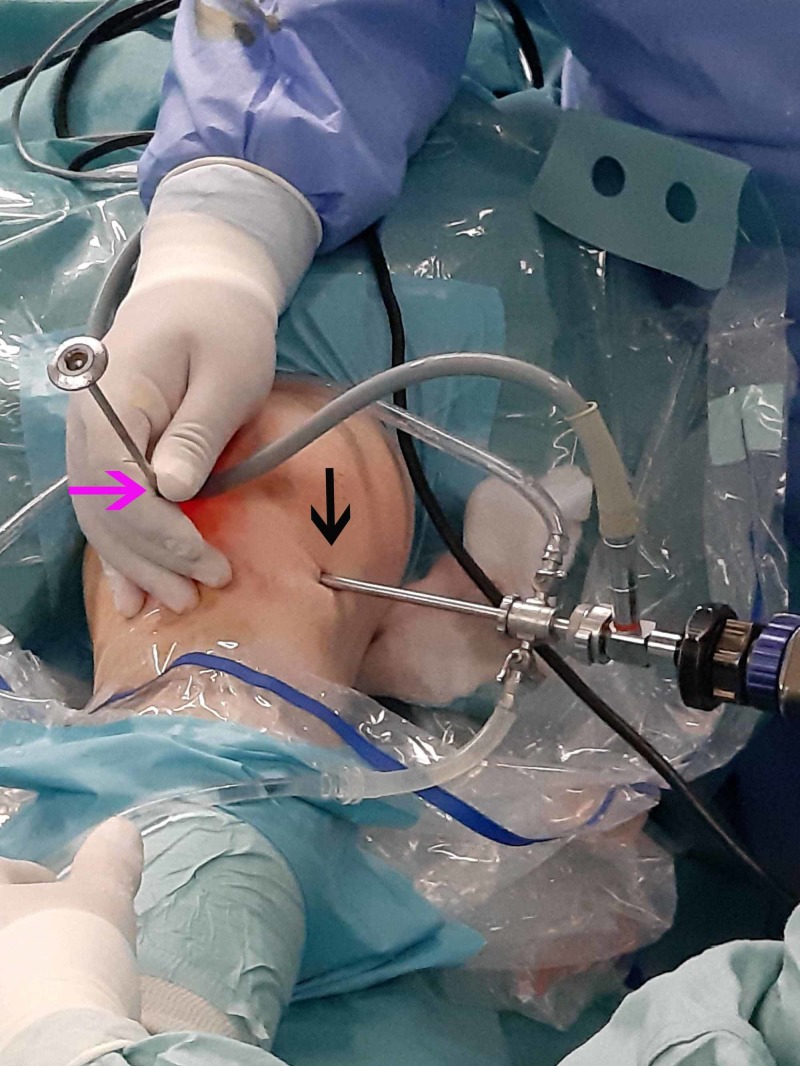
Anteromedial portal A blunt trocar was used to access the tumor from the anteromedial portal (pink arrow), which was established under direct vision from the anterolateral portal (black arrow).

**Figure 7:**
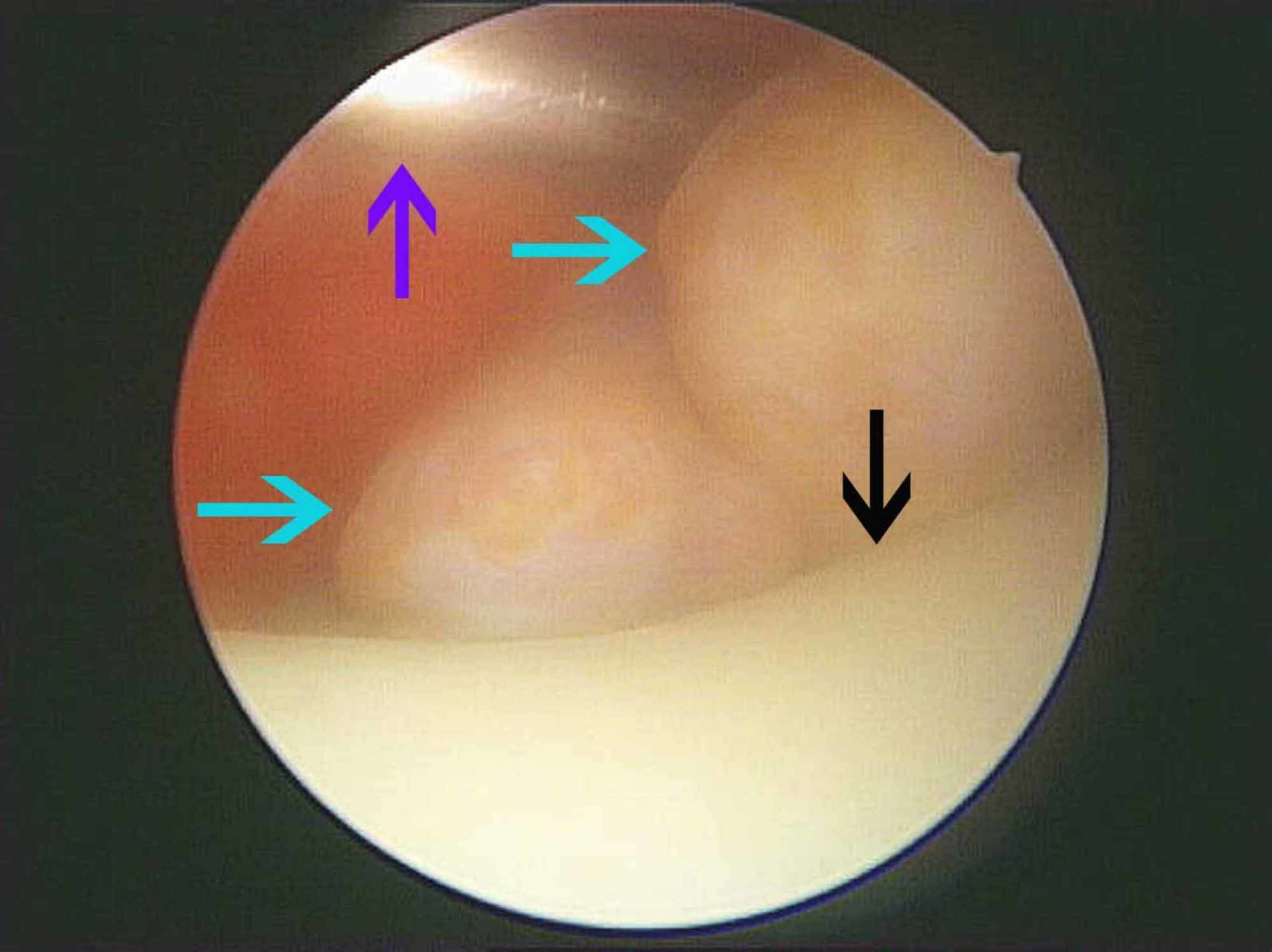
View from the anterolateral portal View from the anterolateral portal (knee extended) where the trocar was inserted by the anteromedial portal (purple arrow). The lateral femoral condyle and trochlea were apparent (black arrow) while the tumor was accessed (blue arrow).

An anteromedial portal was established under direct vision through the anterolateral portal, and a blunt trocar was inserted (Figure 6). The tumor was viewed from the anterolateral portal and accessed through the anteromedial portal (Figure 7).

**Figure 8:**
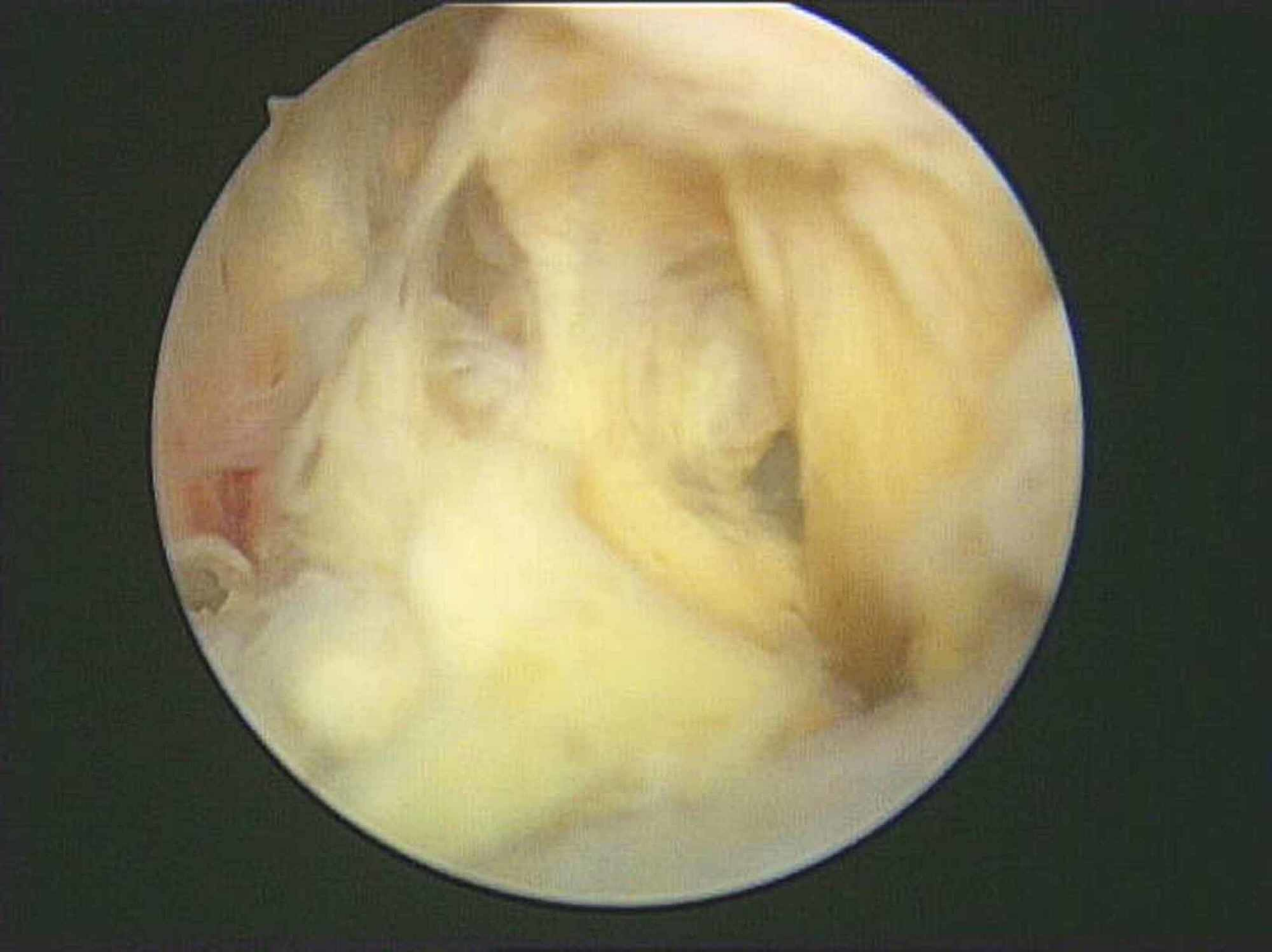
The tumor's attachment

**Figure 9:**
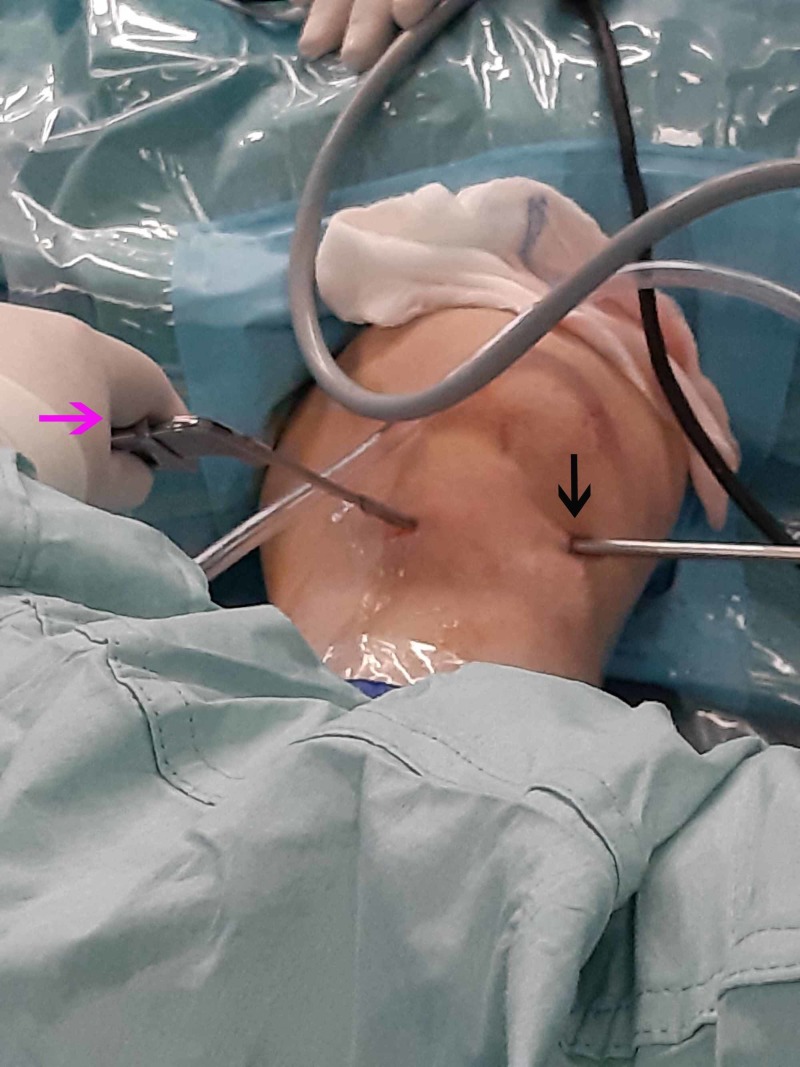
Detachment of the tumor through the anteromedial portal Complete detachment of the tumor was achieved by the anteromedial portal (pink arrow) using a punch while viewing through the anterolateral portal (black arrow).

The tumor’s attachment was also envisaged (Figure 8). The initial detachment of the origin was performed by a blunt trocar. Complete detachment of the tumor was performed using the punch (Figure 9).

 

**Figure 10:**
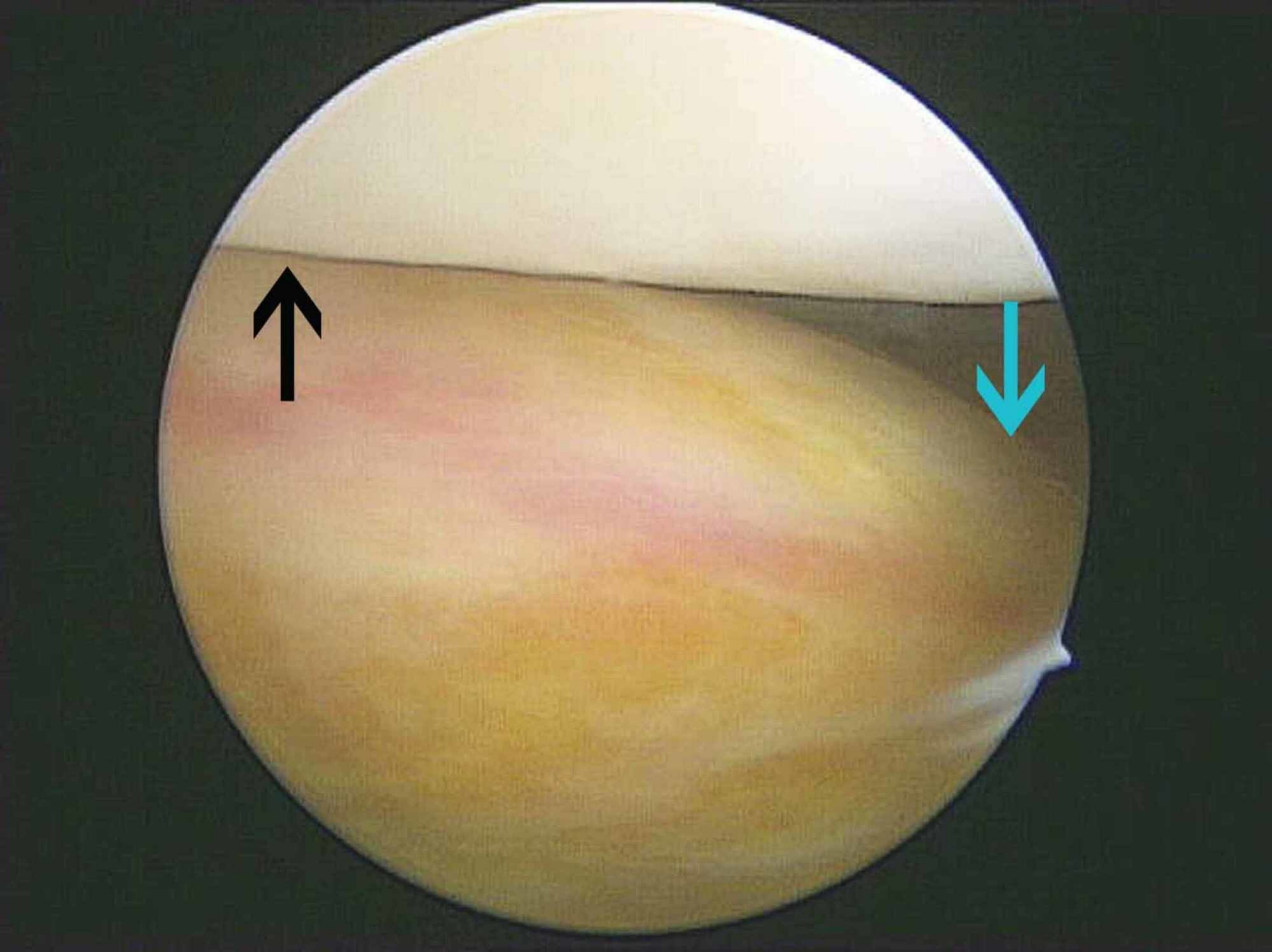
View from the anterolateral portal View from the anterolateral portal after the mass (blue arrow) had been advanced to the suprapatellar pouch. The patella was also evident (black arrow).

**Figure 11:**
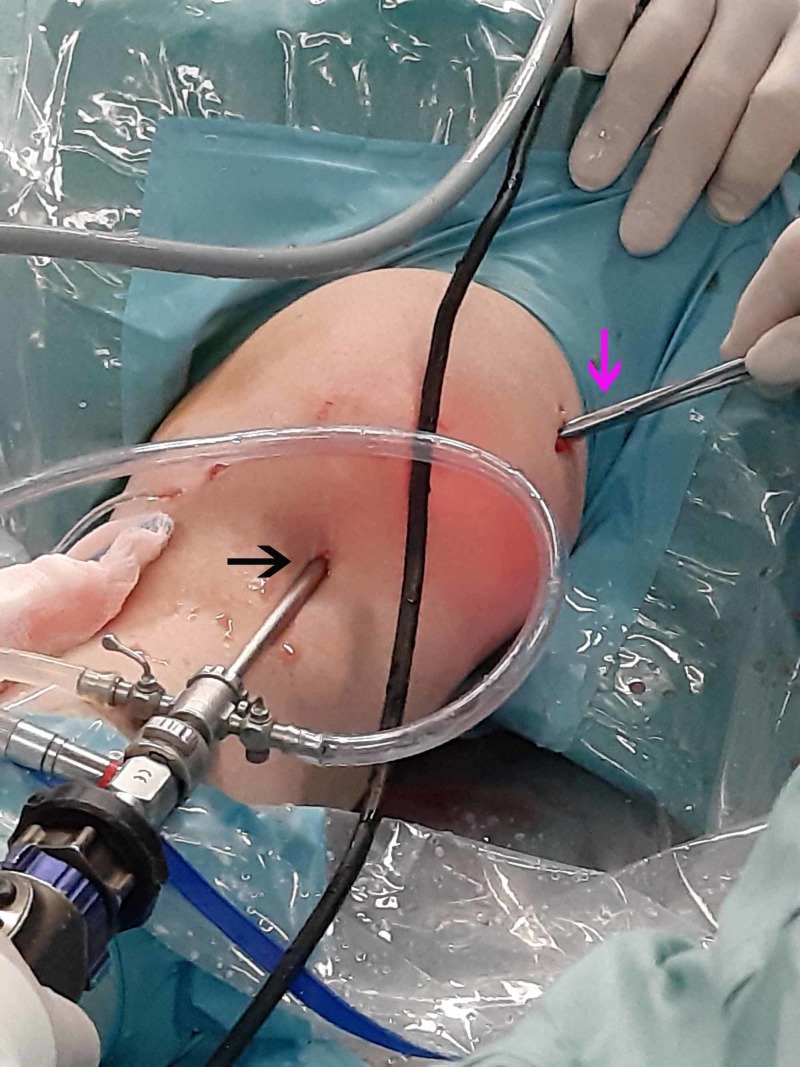
Removal of the tumor The tumor was extracted using a Kocher forceps from the superolateral portal (pink arrow) while viewing from the anterolateral portal (black arrow).

After detachment of the tumor, the mass was advanced toward the suprapatellar pouch (Figure 10) and removed through the superolateral portal after it was enlarged to 2 cm (Figure 11).

**Figure 12:**
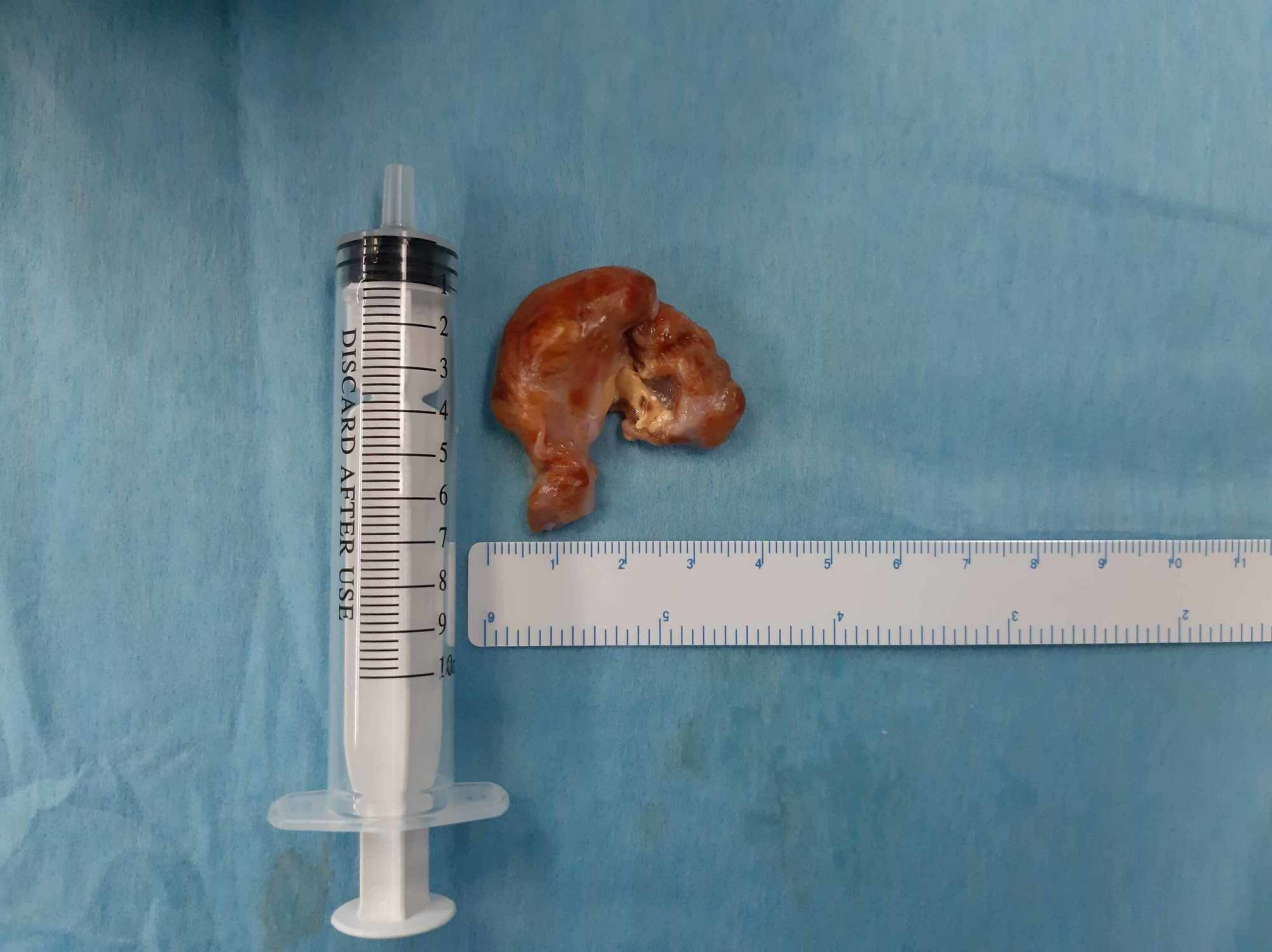
The tumor after extraction The tumor after extraction appearing as a rubbery double-lobed mass sized 4 cm by 3.8 cm.

The tumor after extraction appeared as a double-lobed rubbery mass sized 4 cm by 3.8 cm (Figure 12).

Finally, the attachment of the tumor was cauterized, as well as any other synovial pathology in the lateral gutter of the joint.

The patient had a full ROM of the knee immediately after surgery as there were no complications from the arthroscopic procedure. The tumor was sent for biopsy, and the diagnosis of LPVNS was confirmed. At the six-month follow-up, the patient was symptom-free and had no clinical or radiological evidence of recurrence.

## Discussion

The surgical treatment of LPVNS is generally accepted as either open or arthroscopic localized synovectomy, with no significant difference in recurrence [12]. A large retrospective analysis of 214 cases of knee PVNS, including 100 cases of LPVNS, concluded that there was no difference in local recurrence rates between open and arthroscopic treatment of LPVNS [13]. A review article comparing open with arthroscopic synovectomy for PVNS of the knee including both cases of diffuse PVNS and LPVNS concluded that there was no significant difference between the two in terms of rates of recurrence, complication, and osteoarthritis [14]. In a retrospective study with long-term follow-up, two operative procedures of treating LPVNS, the arthroscopic synovectomy and the arthroscopically assisted mini-open synovectomy, were compared. The authors found no difference between the methods regarding the complication and recurrence rates but suggested that arthroscopically assisted mini-open synovectomy can be used by less experienced surgeons and/or for larger nodules (>3 cm) [15]. In two studies on the arthroscopic treatment of PVNS, there was no recurrence in the with LPVNS treated by arthroscopic local resection [16,17].

The advantages of arthroscopic treatment are the lower rate of complications (e.g., joint stiffness, infections) and quicker functional recovery due to limited invasiveness. In localized forms, resection of the synovial area is generally simple, and the results are excellent and long-lasting. Arthroscopy also allows a more accurate diagnosis and localization of limited lesions [16]. As regards the portals used in arthroscopic synovectomy, it has been reported that two to three portals suffice for PVNS confined to the anterior compartment, whereas five portals are needed for PVNS affecting the anterior and posterior compartments [18]. In one particular case of intra-articular LPVNS with an extra-articular extension through the posterior capsule, the procedure was performed through an all-arthroscopic fashion using four arthroscopic portals (anteromedial, anterolateral, posteromedial, and posterolateral) [19]. Evidence that arthroscopic treatment alone is sufficient for LPVNS is provided by a review of long-term follow-up studies on arthroscopically treated LPVNS that reported no recurrences in patients followed for up to 4.5 years postoperatively [20].

In our patient, arthroscopic resection was possible even though the tumor was particularly large, exceeding 3 cm in both dimensions. This was accomplished using only three portals. There were no procedure-related complications.

## Conclusions

The treatment of LPVNS is the open or arthroscopic complete removal of the mass and local synovectomy. There is no difference in recurrence between open and arthroscopic removal according to the literature. Arthroscopic treatment provides the advantages of a lower rate of complications and quicker functional recovery. When it is confined to the anterior compartment, the removal of LPVNS can be performed through three portals: anterolateral, anteromedial, and superolateral. Arthroscopy is a safe and sufficient treatment for LPVNS when performed accurately, even in the case of large LPVNS tumors > 3 cm. It is, however, technically demanding. We suggest adhering to the method described and following the specific steps proposed in order to avoid complications.

## References

[1] Myers BW, Masi AT (1980). Pigmented villonodular synovitis and tenosynovitis: a clinical epidemiologic study of 166 cases and literature review. Medicine.

[2] Murphey MD, Rhee JH, Lewis RB, Fanburg-Smith JC, Flemming DJ, Walker EA (2008). Pigmented villonodular synovitis: radiologic-pathologic correlation. Radiographics.

[3] O’Connell JX (2000). Pathology of the synovium. Am J Clin Pathol.

[4] Steinmetz S, Rougemont AL, Peter R (2020). Pigmented villonodular synovitis of the hip. EFORT Open Rev.

[5] (2013). WHO Classification of Tumours of Soft Tissue and Bone. WHO, Geneva.

[6] Rydholm U (1998). Pigmented villonodular synovitis. Acta Orthop Scand.

[7] Abdelwahab IF, Kenan S, Steiner GC, Abdul-Quader M (2002). True bursal pigmented villonodular synovitis. Skeletal Radiol.

[8] Tritschler P, Baudrez V, Mutijima E (2020). Diffuse pigmented villonodular synovitis of the subtalar joint. J Belg Soc Radiol.

[9] Fałek A, Niemunis-Sawicka J, Wrona K (2018). Pigmented villonodular synovitis. Folia Med Cracov.

[10] Hantes ME, Basdekis GK, Zibis AH, Karantanas AH, Malizos KN (2005). Localized pigmented villonodular synovitis in the anteromedial compartment of the knee associated with cartilage lesions of the medial femoral condyle: report of a case and review of the literature. Knee Surg Sports Traumatol Arthrosc.

[11] Loriaut P, Djian P, Boyer T, Bonvarlet JP, Delin C, Makridis KG (2012). Arthroscopic treatment of localized pigmented villonodular synovitis of the knee. Knee Surg Sports Traumatol Arthrosc.

[12] Aurégan JC, Klouche S, Bohu Y, Lefèvre N, Herman S, Hardy P (2014). Treatment of pigmented villonodular synovitis of the knee. Arthroscopy.

[13] Patel KH, Gikas PD, Pollock RC (2017). Pigmented villonodular synovitis of the knee: a retrospective analysis of 214 cases at a UK tertiary referral centre. Knee.

[14] Rodriguez-Merchan EC (2014). Open versus arthroscopic synovectomy for pigmented villonodular synovitis of the knee. J Orthop Surg (Hong Kong).

[15] Georgiannos D, Boutsiadis A, Agathangelidis F, Papastergiou S, Karataglis D, Bisbinas I (2017). Arthroscopically-assisted mini open partial synovectomy for the treatment of localized pigmented villonodular synovitis of the knee. A retrospective comparative study with long-term follow up. Int Orthop.

[16] De Ponti A, Sansone V, Malcherè M (2003). Result of arthroscopic treatment of pigmented villonodular synovitis of the knee. Arthroscopy.

[17] Zvijac JE, Lau AC, Hechtman KS, Uribe JW, Tjin-A-Tsoi EW (1999). Arthroscopic treatment of pigmented villonodular synovitis of the knee. Arthroscopy.

[18] Kubat O, Mahnik A, Smoljanović T, Bojanić I (2010). Arthroscopic treatment of localized and diffuse pigmented villonodular synovitis of the knee. Coll Antropol.

[19] Simonetta R, Florio M, Familiari F, Gasparini G, Rosa MA (2017). All-arthroscopic treatment of intra- and extra-articular localized villonodular synovitis of knee. Joints.

[20] Dines JS, DeBerardino TM, Wells JL, Dodson CC, Shindle M, DiCarlo EF, Warren RF (2007). Long-term follow-up of surgically treated localized pigmented villonodular synovitis of the knee. Arthroscopy.

